# Association of MIND diet adherence with micronutrients and executive function among middle‐aged Latinos

**DOI:** 10.1002/alz.087940

**Published:** 2025-01-09

**Authors:** Rafael Guimarães, Yuliana Soto, Kristen DiFilippo, Andiara Schwingel, Susan Aguiñaga

**Affiliations:** ^1^ University of Illinois at Urbana‐Champaign, Urbana, IL USA; ^2^ University of Illinois at Urbana‐Champaign, Champaign, IL USA

## Abstract

**Background:**

By 2060, an estimated 3.5 million Latinos may develop Alzheimer’s disease (AD). Lifestyle factors, such as adhering to the Mediterranean‐DASH Intervention for Neurodegenerative Delay (MIND) diet, may improve cognition and reduce AD risk. This study aims to compare micronutrient intake in low and high MIND diet adherence and assess if higher micronutrient intake is associated with higher executive function (EF) scores in middle‐aged Latinos.

**Method:**

A cross‐sectional study assessed MIND diet adherence and EF in middle‐aged Latinos. The Block 2005 Food Frequency Questionnaire measured diet adherence and micronutrient intake (iron, zinc, calcium, manganese, selenium, Vitamins C, B6, B12, and D). MIND diet scores (<8.5 or ≥8.5) were dichotomized between low‐adherers and high‐adherers. EF was measured using Trails A and B test. A t‐test compared micronutrient intake in low and high adherers. Pearson correlation explored the relationship between micronutrient intake and EF.

**Result:**

Data from 61 participants (Mage = 58.6 ± 8.7, 67% female) show higher micronutrient intake in high adherers. Results indicate a negative correlation between micronutrient intake and Trails A and B test scores (P<.05), suggesting better cognitive performance with higher micronutrient intake for all except Vitamin D. There were significant differences in micronutrient intake between low and high MIND diet adherers for all except Calcium and Vitamin D.

**Conclusion:**

Latinos adhering to the MIND diet show higher micronutrient intake and better EF performance. Future interventions should culturally tailor MIND diet to enhance adherence, potentially improving cognitive function and reducing the risk of AD in this population.

